# Chlorhexidine rinse for prevention of urethritis in men linked to oral sex

**DOI:** 10.1186/1755-7682-3-9

**Published:** 2010-06-11

**Authors:** Jafar Kolahi, Mohamadreza Abrishami, Mohamad Fazilati, Ahmad Soolari

**Affiliations:** 1 Torabinejad Research Center, Isfahan University of Medical Sciences, Isfahan, Iran; 2 Department of Periodontology, Shahid Beheshti University of Medical Sciences, Tehran, Iran; 3 Department of Food Sciences, Isfahan University of Technology, Isfahan, Iran; 4 PEARL Program, College of Dentistry, New York University, NY, USA

## Abstract

**Background:**

Oral sex among teenagers is on the rise. Similarity between the oral flora and organisms recovered from nongonococcal urethritis and prostatitis, points to retrograde entry of bacteria from oral cavity into the urethra following insertive oral intercourse.

**Presentation of the hypothesis:**

Chlorhexidine has a wide spectrum of anti-bactericidal activity encompassing gram positive and negative bacteria. It is also effective against HIV and HBV. It produced large and prolonged reductions in salivary bacterial counts within 7-h of its use. Hence, it would seem logic to postulate that rinsing with chlorhexidine before oral sex will be effective for prevention of retrograde entry of bacteria from oral cavity into the urethra. The recommendation for rinsing will be: 15 ml of a 0.12% or 10 ml of 0.2% chlorhexidine rinse for 30 seconds. Also other drug delivery systems such as chlorhexidine chewing gum or spray can be used.

**Testing the hypothesis:**

Men suffering from recurrent nongonococcal urethritis or prostatitis are good subjects for testing the hypothesis. They perform genital safe sex via consistent use of condom. Yet they generally received unprotected insertive oral intercourse. Chlorhexidine can be used for prevention of recurrences of the disease.

**Implications of the hypothesis:**

The chlorhexidine will be a new, easy, attractive and effective method for reduction of nongonococcal urethritis, prostatitis and epidydimitis following insertive oral intercourse. It is poorly absorbed from skin, mucosa and gastrointestinal tract indicating systemic safety of chlorhexidine. The agent does not cause any bacterial resistance and supra-infection.

## Background

The first comprehensive study to examine the prevalence of the oral sex was published in the Time magazine [1]. The report by the National Center for Health Statistics was based on a computer-administered survey of over 12,000 Americans between the ages of 15 and 44, and stated that over half of the teenagers questioned have had oral sex. This report provided evidence that oral sex among teenagers is "on the rise".

However, direct and indirect evidence from studies of patients with nongonococcal urethritis and prostatitis points to retrograde entry of bacteria from the oral cavity to the urethra following oral intercourse as primary mode of transmission for pathogenesis of infection [2-6]. Evidence is based upon similarity of organisms recovered from infection and normal oropharyngeal flora such as Streptococci, Haemophilus species, N. meningitides, Adenoviruses and HSV-1 [4].

Nevertheless, if sexually transmitted disease (STD) status is unknown for partners, condoms or dental dams is recommended when performing or receiving oral intercourse. The plastic wrap may also be used as a barrier during oral intercourse, but many find that the thickness of the plastic dulls sensation [7].

The aim of this hypothesis is to suggest a new, easy, attractive and effective method for prevention of nongonococcal urethritis, prostatitis and epidydimitis following oral intercourse.

## Presentation of the hypothesis

Chlorhexidine is a bisbiguanide antiseptic which has a wide spectrum of bactericidal activity encompassing gram positive and gram negative bacteria. It is also effective against some fungi and yeast, including Candida, and some lipophilic viruses including HIV and HBV [8]. Chlorhexidine (C_22 _H_30 _Cl _2_N_10_) is a strong base and dicationic at pH levels above 3.5. The bactericidal effect of chlorhexidine is due to the cationic nature of the agent binding to extra microbial complexes and negatively charged microbial cell wall, thereby altering the cells osmotic equilibrium [9].

Of more interest, the usage of 0.12% or 0.2% commercial formulations of chlorhexidine mouthwashes produces a large and prolonged reductions in salivary bacterial counts within the 7-h period [10]. Hence, it would seem logic to postulate that prophylactic rinsing with chlorhexidine before oral intercourse will reduce retrograde entry of bacteria via the urethra. The recommendation for rinsing is 15 ml of a 0.12% or 10 ml of 0.2% oral chlorhexidine rinse for 30 seconds [8]. The previous published date recommended 2 hour post brushing to optimize anti-microbial activities of chlorhexidine rinse [8]. Also other drug delivery systems such as chlorhexidine chewing gum [11] or spray [12] can be used.

In the same way, chlorhexidine have been used successfully for controlling the oropharyngeal flora and accordingly prevention of the ventilator-associated pneumonia involving aspiration of bacteria from the oropharynx into the lung [13,14].

## Testing the hypothesis

Men suffering from recurrent nongonococcal urethritis or prostatitis are good subjects for testing the hypothesis. They perform genital safe sex via consistent use of condom. Yet they generally received unprotected oral intercourse. Chlorhexidine can be used for prevention of recurrences of the disease.

## Implications of the hypothesis

The chlorhexidine rinse will be a new, easy, attractive and effective method for reduction of nongonococcal urethritis, prostatitis and epidydimitis following insertive oral intercourse. It is poorly absorbed from skin, mucosa and gastrointestinal tract indicating systemic safety of chlorhexidine. The agent does not cause any bacterial resistance and supra-infection.

Current literatures [2-6,8] support prophylactic use of chlorhexidine rinse as a safe and effective way for reduction of retrograde entry of bacteria from oral cavity to the urethra following insertive oral intercourse via large and prolonged reductions of the oral bacterial counts. This approach will be a new, easy, attractive and effective method for prevention of nongonococcal urethritis, prostatitis and epidydimitis following insertive oral intercourse.

Oral health professionals have used chlorhexidine for over three decades. It is poorly absorbed from skin, mucosa and gastrointestinal tract [8,15] indicating systemic safety of chlorhexidine. The agent does not cause any bacterial resistance and supra-infection following long term oral usage [8].

Despite the excellent anti-microbial property of chlorhexidine, clinical usage of the agent is limited by the possible local side effects including: 1) reversible staining of the teeth and tongue 2) transient taste perturbation 3) oral mucosal erosion 4) occasional parotid gland swelling and 5) increases supra gingival calculus formation [8]. The most common and problematic side effect is reversible staining. Staining is mild and visible within a week and becomes moderate in 50% of patient after six months following continues usage of chlorhexidine rinse each 12 h. The long term use of chlorhexidine rinse is associated with heavy reversible staining. The staining is more pronounced in patients who have heavier accumulations of plaque [8]. The long term use of chlorhexidine rinse for the purpose of reduction of STD has not been evaluated yet.

## List of abbreviations

STD: Sexually transmitted disease.

## Competing interests

The authors declare that they have no competing interests.

## Authors' contributions

Main idea of this hypothesis is provided by JK. He wrote the main draft of manuscript. MA, MF and AS helped to consider clinical aspects of the hypothesis. All authors read and approved the final manuscript.
